# Why men with a low-risk prostate cancer select and stay on active surveillance: A qualitative study

**DOI:** 10.1371/journal.pone.0225134

**Published:** 2019-11-20

**Authors:** Aaron T. Seaman, Kathryn L. Taylor, Kimberly Davis, Kenneth G. Nepple, John H. Lynch, Anthony D. Oberle, Ingrid J. Hall, Robert J. Volk, Heather Schacht Reisinger, Richard M. Hoffman

**Affiliations:** 1 Department of Internal Medicine, University of Iowa Carver College of Medicine, Iowa City, IA, United States of America; 2 Cancer Prevention and Control Program, Lombardi Comprehensive Cancer Center, Georgetown University, Washington, DC, United States of America; 3 Department of Urology, University of Iowa Carver College of Medicine, Iowa City, IA, United States of America; 4 Holden Comprehensive Cancer Center, University of Iowa, Iowa City, IA, United States of America; 5 Department of Urology, Georgetown University, Washington, DC, United States of America; 6 Prostate Cancer Center, Lombardi Comprehensive Cancer Center, Georgetown University, Washington, DC, United States of America; 7 Epidemiology and Applied Research Branch, Division of Cancer Prevention and Control, Centers for Disease Control and Prevention, Atlanta, GA, United States of America; 8 Department of Health Services Research, Division of Cancer Prevention and Population Sciences, University of Texas MD Anderson Cancer Center, Houston, TX, United States of America; University of Sussex, UNITED KINGDOM

## Abstract

**Objective:**

Active surveillance (AS) is an increasingly utilized strategy for monitoring men with low-risk prostate cancer (PCa) that allows them to defer active treatment (AT) in the absence of cancer progression. Studies have explored reasons for selecting AS and for then switching to AT, but less is known about men’s experiences being on AS. We interviewed men to determine the clinical and psychological factors associated with selecting and adhering to AS protocols.

**Methods:**

We conducted semi-structured interviews with men with a low-risk PCa at two academic medical centers. Subjects had either been on AS for ≥ 1 year or had opted for AT after a period of AS. We used an iterative, content-driven approach to analyze the interviews and to identify themes.

**Results:**

We enrolled 21 subjects, mean age 70.4 years, 3 racial/ethnic minorities, and 16 still on AS. Men recognized the favorable prognosis of their cancer (some had sought second opinions when initially offered AT), valued avoiding treatment complications, were reassured that close monitoring would identify progression early enough to be successfully treated, and trusted their urologists. Although men reported feeling anxious around the time of surveillance testing, those who switched to AT did so based only on evidence of cancer progression.

**Conclusions:**

Our selected sample was comfortable being on AS because they understood and valued the rationale for this approach. However, this highlights the importance of ensuring that men newly diagnosed with a low-risk PCa are provided sufficient information about prognosis and treatment options to make informed decisions.

## Introduction

Active surveillance (AS) is a monitoring strategy for men with low-risk prostate cancer (PCa) that allows men to defer active treatment (AT) in the absence of cancer progression. The advent of PSA testing in the late 1980s was associated with a dramatic increase in the number of men being diagnosed with a low-risk PCa.[1] For many years the majority of men with a low-risk PCa were aggressively treated with surgery or radiation, even though treatments potentially could result in more harms than benefits.[2–4] A 2011 National Institutes of Health State-of-the-Science (SOS) Conference Statement on the Role of Active Surveillance in the Management of Men With Localized Prostate Cancer concluded that more men should be offered the option of AS.[5, 6] Panelists highlighted the need for further research to determine factors associated with offering, accepting, and adhering to AS, the need to develop and evaluate optimal protocols for AS, and the importance of better supporting patient decision making.

Professional society guidelines now uniformly recommend that men with a low-risk PCa consider AS.[7, 8] Observational data suggest that uptake of AS is increasing, though each year about 9% of men on AS switch to AT.[9–12] Additionally, studies suggest that men may not be adherent with monitoring protocols, particularly in obtaining surveillance biopsies.[13, 14] While AS may mitigate the harms of overdiagnosis, lack of adherence with surveillance monitoring could lead to more men progressing to advanced-stage, incurable disease.

A meta-analysis cited numerous cohort studies that quantitatively evaluated the reasons for switching from AS to AT, generally classifying them as either due to clinical progression or the non-specific category of patient preference/anxiety.[15] An estimated 20 percent of treatment changes were due to non-clinical patient factors. We found a number of studies that qualitatively evaluated psychological factors associated with selecting and adhering to AS, which could potentially shed light on the non-clinical factors.[16–24] However, none of these studies included both men who had remained on AS and underwent surveillance monitoring for at least a year, (the time period conventionally used to define whether a man has deferred active treatment),[10, 12] and those who switched from AS to AT. In this study, we interviewed men from two academic medical institutions who had initially selected AS to determine the clinical and psychological decisional factors associated with initial selection of and adherence to AS protocols.

## Methods

### Study setting and sample

We conducted in-depth, semi-structured interviews with men who had been on AS for at least one year. Patients who opted for AT after a period of AS were also eligible. All but two participants, who had MRI surveillance only, had undergone at least one surveillance biopsy. Participants were recruited through urology clinics at the University of Iowa Hospitals and Clinics (UIHC) and Georgetown University Medical Center (GUMC). The Hawk IRB at UIHC (201708714) and the Georgetown MedStar IRB at GUMC (2017–1061) approved the study. Subjects provided oral consent to participate in the study.

### Data collection

Our interview guide questions were designed to elicit participants’ 1) experiences of cancer diagnosis and treatment discussions, 2) perceived decisional influences when choosing AS, 3) experience with AS monitoring protocols, 4) attitudes toward AS and living with PCa, and 5) future intentions. The interview guide, designed to be semi-structured,[25] drew from both the active surveillance literature and our previous research examining selection factors,[26] while leaving open the potential for participants to bring up topics and insights they felt were central to their experience of active surveillance. For participants who opted for treatment after a period of AS, we asked about the factors that influenced that decision.

We conducted interviews between September 2017 and February 2018. Urologists identified eligible patients at each institution. At UIHC, letters explaining the study were sent to eligible patients, along with a self-addressed, stamped postcard that could be returned if patients did not want to participate. At GUMC, a urologist approached patients and forwarded contact information of those interested to researchers. We asked patients who did not opt out if they would like to participate. We reviewed the interview procedure with willing participants and obtained oral consent. Research team members (AS at UIHC, KT and KD at GUMC), have PhD training in qualitative research, are experienced interviewers, and had no previous relationship with the participants before conducting the interviews. All investigators hold faculty appointments in their respective institutions. AS is a male, KT and KD are females. The research team has previously collaborated on cancer survivorship studies, particularly among men with prostate cancer, with a focus on decision making. Interviews were conducted in person or by phone, lasted approximately one hour, and were audio recorded. Participants received a $75 gift card and a parking voucher for clinic interviews.

### Data analysis

A third-party transcription service transcribed interviews which were then reviewed for accuracy and uploaded to MAXQDA 18^™^ (VERBI GmbH), a qualitative data management software. We then conducted a thematic analysis of the interviews, using an iterative, content-driven approach that utilized both deductive and inductive coding.[27] Team members initially hand coded 3 transcripts, using an open-coding process that allowed for an inductive data examination. Members met to discuss codes, resolved any discrepancies, and compiled a preliminary codebook based on both inductive and deductive codes, derived from prior research and extant literature. We collectively hand coded 2 additional transcripts with the preliminary codebook to ensure it was comprehensive. Two members coded the remaining 16 transcripts in MAXQDA, entered the coding from the five hand-coded transcripts, and prepared a detailed summary document of thematic domains, codes, and quotations, which was circulated to the entire team. We stopped recruitment once we achieved data saturation. We did not return transcripts to participants for comment and/or correction. We did not seek feedback on our findings from the participants.

## Results

### Participant characteristics

We contacted 30 potential subjects in Iowa, approaching 12 subjects in clinic and conducting 11 interviews. We contacted 13 potential subjects at Georgetown, approaching them all in clinic and conducting 11 interviews. Overall, we completed 22 interviews, 14 by phone and 8 in person in the clinic. Twenty-one participants were included in the final analysis; one was excluded after we determined that he was never on AS. While the interviews were being conducted, 16 participants were still on AS while five had switched to AT (4 radical prostatectomy, 1 external beam radiation therapy). Table 1 presents participant demographic information. Table 2 presents thematic domains and representative quotations (see also S1 Patient Interview Summary).

**Table 1:** Participant characteristics.

Characteristic	Mean (range) or N (%)
Age (years)	70.4 (56–84)
Race/Ethnicity	
White	18 (86)
Black	2 (10)
Other	1 (5)
Marital Status	
Spouse/partner	18 (86)
Years since diagnosis	3.9 (1.25–10)
Treatment status	
Active Surveillance	16 (76)
Active Treatment	5 (24)

**Table 2:** Thematic domains and representative quotations.

Decision Factors for Selecting Active Surveillance	
Presentation of AS	My wife and I met with [the urologist], and he had suggested waiting and watching and seeing ‘cause I was still quite young. …He said, “Every six months you’ll come in for testing, and we’ll watch it that way.” I was really afraid of surgery ‘cause I really don’t like the incontinence and other things that can happen. [The urologist] sent me down to [the physician] in charge of the CyberKnife surgery [sic]. He met with me for about an hour that day, …explained to me at that young age he would go for surgery, not CyberKnife ‘cause they don’t have a 20-year study yet to what happens to you down the line but, you know, it’s still my choice. (GU06, age 65)
	He said we could look at it in six months and keep an eye on it. He might have said, at one point or another, that there are a lot of ways to treat it other than surgery. I felt that letting six months go, it wasn’t gonna ravage me and it wouldn’t be too late. I didn’t worry about that. (GU05, age 79)
	The way it was presented to me was that we caught it at such an early stage. It wasn’t really addressed to do it any other way for a time other than just to go on AS, then we’ll see how it goes. …I mean nobody was pushing, or suggesting or anything other than that. …There wasn’t any emphasis put on doing anything else. (GU07, age 75)
	As I say, there wasn’t a choice given to me. This is just what he advised. (UI03, age 74)
	It was like line me up for the surgeon. …Just his attitude, I was real concerned about that. (UI11, age 71)
Trust in Urologist	I know that [my urologist] is at the top of the ladder…. And I just follow pretty much what he says. He wouldn’t be telling me, in my opinion, if he didn’t need to do it. (GU13, age 84)
	I had faith in my doctor. After meeting [my urologist], and what he was telling me about it, and the surveillance, and just monitoring it with the exams and the PSA, I just agreed with it. (UI09, age 56)
Confidence in AS	Now, if it would have been Gleason seven or eight and aggressive, I would have had a whole different attitude. …I think because I felt comfortable that it was a low grade, not aggressive, I felt I have some time. (UI06, age 68)
	I thought as long as we were keeping an eye on it. I got the impression that for anything to drastically happen would be very, very small, small miniscule chance of that happening, especially if I was going there three times a year, once every four months for a PSA. We're keeping a really good eye on it. We're doing what we need to do. (UI09, age 56)
	I think he’s cautious enough that if he thought we should biopsy it or consider doing something right away, he would’ve said so. I trusted his—and I believe prostate cancer, in general, is fairly slow-growing, so it just made sense to me. (GU05, age 79)
Uncertainty Tolerance	Maybe you can call me crazy. …I feel very confident in the approach that I’m on with [my urologist]. …I haven’t experienced anxiety. I mean, it hasn’t really distracted [me] for more than a second, occasionally, from my normal routine. …I know some people that worry. I’ve just never been that way. (GU03, age 77)
	I think my own personality played a role. I’m very good at ignoring things if I want to. During the period of AS, I didn’t really pay that much attention. It was okay, six months from now, I gotta get another test. I’d start thinking about it, and there’d be a little anxiety in the week or two before, but I really didn’t think very much about it in between. (UI02, age 74)
Active Surveillance Protocols	
Biopsy Schedule	I had nice protocols. When I left every doctor’s appointment, “All right. I wanna see you back in three months.” And [my urologist] was always, “Get it as close as you can. I know your schedule is a mess, but, you know.” …And I think I would was never off more than a week either way. So yeah, it was pretty good. (GU04, age 58)
	When they do the PSA test, I’m assuming if I’m having more issues they’ll look in a little deeper. …Depending on what they find out in the PSA, then they’ll determine if I have to have a biopsy, but I believe he told me I probably have a biopsy every year or two (UI08, age 62)
	“I wouldn’t have entertained [a repeat biopsy] particularly, unless there was an awfully good reason” (GU14, age 79).
Use of MRI	Now, I don’t know anything about this case as far as how rapid things accelerate, but in my own mind, I guess, I’m not particularly alarmed, concerned, disturbed. I mean, I had a biopsy 18 months ago. I had this MRI in December of last year, ten months ago. Those things, which to me, I’m attaching a lot more significance to, maybe unnecessarily so, than those PSA test numbers. (GU03, 77)
	Then when they said, “Oh yeah, we’re gonna get this—what is it—fused MRI here in the first of the year.” I said, well yeah, that seems to be what everybody’s swearing by as far as helping pinpoint things as well as making certain you hit the right spot for checking for things. I went into the spring here with an idea as far as well, we’re gonna find it for certain, because we’re not shooting blind anymore, a little bit better at least, and after all…I didn’t have anything that they were finding, showing up, which was one heck of a relief. …I thought we used the MRI as a tool to decide now is the time to do another check, because supposedly we found a hotspot. As a result, I would say it would hopefully help eliminate unnecessary biopsies. (UI11, 71)
Experiences of Active Surveillance	
Importance of quality of life	I’m looking for quality of life—I don’t think, right at this point, it’s gonna kill me, but if I have the prostate taken out, and then I have all these other issues, my quality of life. …That’s why the active surveillance to me—it’s okay for me right now (UI08, age 62)
Treatment inevitability	You know you’re gonna have to treat it at some point, or you should. You’re gonna have to deal with it. (GU04, 58)
Confidence in protocol and urologist	I'm glad we're under active surveillance, because that means, if there's any change, he would know quickly. I think it sort of means they will know as soon as possible. (GU02, age 78)
Active engagement	I feel good that I’m taking steps to monitor this thing. (UI05, age 68)
	I think part of the active surveillance—I mean, if you’re doing surveillance, you are watching for issues to happen. I mean, that’s part of the definition of the word” (UI07, age 67).
	I feel very positive about doing it. I don't think it is exactly, but I almost consider it a treatment. It's not that it's actually intervening in some way. …[But] based on the biopsy, we'll treat it accordingly. …I mean, what's happening to me might be mostly diagnostic in the true sense of medicine, but from the patient's standpoint, I'm considering it part of the modality. This is what you do in order to remain healthy. (GU01, age 69)
Routine nature of AS	I just got comfortable with the security of being on surveillance. …I just got to the point where that’s not a major factor. There was no depression from that, or concern or worry about it. In the beginning once I found out I had cancer, every problem I felt was related. I’d get a sore shoulder or whatever—so you’re very concerned. I’m out of that stage now” (GU10, age 72).
	I guess I don’t look at it as a surveillance program. I’m not waiting for my next doctor appointment to find out that I have prostate cancer or not. I don’t see that as an issue. It’s just that I went, the last biopsy was fine. Everything’s fine. I’ll just watch my diet and move on in life here. (UI03, age 74).
Social support	I keep everybody involved, because I think if you don’t, you get into a situation where you’re moping. If it’s bugging you, you can talk to somebody about it. Then most likely you can process it and get it out of your system a little quicker. (UI08, age 62).
	Well, my mother and father. They're still with me. They're 86 and 84 right now. They thought it was a good idea. A good move. Because I don't think they ever like to see their children go on the operation table. My brother. My surviving sister. I had three sisters, but I've lost two of my sisters. She was very supportive. My two daughters were very supportive. At the time, the gal I was dating was very, very supportive. (UI09, age 56)
	She makes sure I actually go for the tests every six months. …Then, as soon as the letter comes in the mail, I have to rip it open immediately. (GU06, age 65)
	She thought it was important for us to have a spreadsheet and monitor our results of all our examinations. Our blood pressure, our heart rate, or whatever else we have. She has a spreadsheet and we have the PSA on it. She says, “Okay, what’s your PSA?” I tell her. What’s my blood pressure and all that and so I tell her and she posts into a Spreadsheet. It seemed like before my next appointment, she pulls that out and says, “Okay, here’s—have this in your head.” (UI05, age 68)
Intentions for Continuing Active Surveillance and Considerations for Switching to Active Treatment	
Intentions for AS	As long as I know there’s still cancer in there and as long as it isn’t bothering me or being a high-risk situation, I have no intent whatsoever to change. (UI07, age 67).
	I think I would stay with active surveillance, you know, from the old adage if ain’t broke, don’t fix it, or don’t try to fix it. And, in the event that there’s a sudden change, then certainly I’d have to reconsider what the options are at that time. (GU14, age 79)
Tumor progression	We decided on a game plan, and how to go about it, and keep an eye on it. We kept an eye on it for a good year. Then the following year, we did a second biopsy. The second biopsy revealed that there was another spot. It looked like it had moved. …That's when we made a decision to go ahead and have it removed. (UI09, age 56)
	I will continue until it starts progressing. If it progresses then I’ll do something about it. …Yeah, I’m gonna continue until it creates a danger to me. I’m not in any danger right now. (UI08, age 62)
Age	[*when considering ending AS*] Unless I get so old that I don’t think it’s important anymore. That would probably be at least 20 years from now. I intend to live a long time. (UI07, age 67)
Treatment advances	Part of the reason, I figure if I can get by on active surveillance without it killing me, God knows what they’re gonna come up with six months from now, where they’re making advances every day. …Maybe they’re gonna come up with a pill I can take and shrink the prostate and cure the cancer. (UI06, age 68).
Potential for regret	I look back, and I got another two more years basically [without treatment and its side effects], and I’m really lucky to have that because of where I’m at now. (UI10, age 66)
	I had not realized that PSA tests weren’t perfectly accurate, but a sudden spike followed by a drop was something that I hadn’t really anticipated. And what I thought about afterwards was, if those two tests had been in the reverse sequence, I wouldn’t have had surgery. I would never have gone that far. I would have thought things were just going along. I am not sure, in a similar circumstance, whether I would go with surveillance again. Not because it wasn’t the right decision, but because I have had the experience of what happens when the right decision doesn’t lead to the right results. And it has made me more aware that even when you make the best possible decision based on the best possible information, things can go wrong. …[H]aving gone through this, I think were a similar situation to arise in the future, I would put more weight on taking action than on waiting and watching. …I might be more likely to be one of those people who would say, “Get it out.” (UI02, age 74)

### Decision factors for selecting active surveillance

Participants reported multiple factors contributed to initially selecting AS. All wanted to avoid treatment side effects, particularly sexual and urinary dysfunction. A participant’s relationship with his urologist and personal characteristics, such as information-seeking tendencies and a tolerance of uncertainty, all affected his willingness to consider AS.

Participants described their initial treatment conversations with urologists, the range of their information needs, and urologists’ presentation styles. Treatment discussions with urologists ranged from in-depth conversations addressing the severity of their PCa, what AS entailed, and treatment options, to more directive guidance with limited options presented. About half of the men described conversations in which urologists presented information about either treatment options or AS, but not both. Four reported that the diagnosing urologist did not discuss AS at all, describing their unease at what they felt was a treatment decision rushed by their urologist. They also described themselves as active information seekers, asking questions of their clinicians and consulting print and online sources, which led them to seek second opinions.

Participants discussed numerous factors contributing to their confidence in selecting AS. Trust in their treating urologists was a primary factor. Sometimes trust resulted from an impression of a urologists’ prominence, while some participants talked about trust engendered through personal contact. Conversely, lack of trust—whether resulting from an aggressive recommendation, lack of confidence in expertise, or another factor—was a reason to seek a second opinion. Participants also expressed confidence in their diagnosis as low-risk and surveillance as effective. Results from diagnostic testing were compelling, with participants reporting they might have chosen differently had their PSA levels or Gleason scores been higher. Participants also talked about their confidence in the surveillance process to detect any changes in their PCa, seeing frequent testing as comforting. Often trust in their urologist and confidence in surveillance buoyed each other. Finally, some participants described themselves as tolerant of uncertainty, reporting that they were generally not anxious about their health.

Participants discussed undertaking a range of information-seeking activities when deciding about AS. While some received educational materials from the urologist, most who sought additional information turned to online sources and research articles. One mentioned reading a popular book on PCa. Several referenced friends with PCa, often citing the friend’s post-treatment side effects as a factor in their selecting AS. No one remembered being provided a decision aid to guide the treatment discussion.

### Active surveillance protocols

Participants all had regular PSA testing and digital rectal exams, with frequency ranging from one to four times a year, though biopsies were performed less systematically. Two participants had not had any biopsies since their diagnosis, while another had annual biopsies, stating that the biopsies were “never more than a week off either way.” Most had undergone 1 or 2 biopsies since diagnosis, approximately every 1 to 2 years. Sometimes, patients and urologists agreed upon a flexible biopsy schedule, depending on other factors such as PSA levels. A few mentioned wanting to avoid biopsies, preferring the alternative of surveillance MRI tests. MRI-guided biopsy results were perceived by some as being more reliable and reassuring than results from PSA tests or a biopsy performed only with ultrasound.

### Experiences of active surveillance

Several participants mentioned avoiding AT as a primary benefit of AS because they wanted to avoid potential side effects, especially urinary incontinence and loss of sexual function. They discussed the importance of preserving quality of life, though seeing AS as just temporarily delaying the inevitable.

Participants reported psychological benefits from AS. They were reassured by being closely monitored, feeling that any progression in their PCa would be detected in time to offer curative AT. Some participants described a sense of engagement in their own care through AS. Others discussed that the routinizing process of AS, its regularity, led to increased comfort with living with cancer. Those who described feeling anxiety did so only in the days leading up to an appointment and stated that it quickly dissipated after they received their results.

Participants also appreciated having flexibility in the surveillance protocol, especially in terms of scheduling biopsies. Patients and urologists were able to determine a schedule that provided the level of surveillance both felt was appropriate.

Along with confidence in the surveillance protocol, participants reported that trust in and support from their urologist continued to be an important factor in their comfort with AS. Participants also talked about positive social support from their spouses, other family members, and friends, even though they sometimes felt it necessary to initially justify their decision. Our subjects reported hearing concerns that not aggressively treating their cancer, but they were also aware of negative experiences of men in their social network who regretted aggressively treating their cancers due to treatment complications or long-term side effects. Usually, once family and friends understood that the cancer was not life-threatening, they could appreciate the importance of avoiding unnecessary treatment. Support could be emotional or more instrumental support, as some participants described spouses who attended appointments, were centrally involved in decision making, or helped keep track of health information.

### Intentions for continuing active surveillance and considerations for switching to AT

All participants who were on AS at the time of the interview intended to stay on AS. Participants did describe, however, a number of reasons why they might revisit their decision for AS and consider treatment. In addition to tumor progression, participants discussed their urologists’ recommendations, urinary symptoms or other health considerations, treatment advances, and their own advancing age as potential factors. No one discussed anxiety or uncertainty about being on AS as a reason to switch to AT.

Among those participants who underwent treatment after a period of AS, all five reported choosing treatment in light of evidence that their tumor had progressed and at their urologist’s recommendation. Despite having to eventually switch to an AT, most participants said they did not regret the initial choice of AS. They described it as giving them more time free from treatment side effects. The one participant who did express some potential regret about waiting to treat described it in terms of hindsight, discussing that he might not make the same decision a second time, if he had the knowledge he did now, because “I have had the experience of what happens when the right decision doesn’t lead to the right results.”

Participants who were still on AS also reported feeling that tumor progression and urologist recommendation would be the most influential factors in a decision to treat. Of the participants who also were experiencing urinary symptoms, some felt that worsening of these symptoms could cause them to consider treatment. Others discussed that they might reconsider if there were advances in treatment. A few people also noted that their age could influence their decision to stop AS, describing reaching an age where they felt that treatment would no longer be beneficial.

## Discussion

We qualitatively evaluated men diagnosed with a low-risk PCa who initially opted to manage their cancer with AS and were being followed in academic medical centers. Men were comfortable with AS because they recognized the favorable prognosis of their cancer, valued avoiding treatment complications, were reassured that close monitoring would identify progression early enough to be successfully treated, and trusted their urologists. Those who switched to AT did so based on evidence of cancer progression, but not for anxiety. Our findings have implications for assisting men during the treatment decision-making process.

While men generally reported that their cancers were classified as low-risk based on routine clinical measures, including PSA, digital rectal examination, and Gleason scores, some also underwent MRI for staging and/or biopsy guidance. MRI results were considered more reassuring because other tests, particularly PSA values, were thought to be potentially unreliable. Previous studies have also shown men questioning the validity of clinical measures alone[21] and some AS protocols require a second biopsy to confirm risk level.[7] Increasing the acceptance of AS may require incorporating additional modalities, such as MRI imaging, MRI-guided biopsies, or genomic tests into risk assessments.[7]

Men understood that a low-risk PCa was unlikely to progress quickly if at all and did not require immediate treatment. While most received this information from the diagnosing urologist, several men had sought out second opinions at the academic medical center because they initially were offered only AT. However, many men had researched PCa treatment options and were aware that AS was appropriate for their low-risk cancer. Other investigators have shown that barriers to selecting AS include being unaware of risk status and not having been offered the option.[24, 28, 29] This suggests the importance of providing all newly-diagnosed men with comprehensive information about cancer prognosis and treatment options, as recommended by the 2011 SOS Conference.[6] Given the complex tradeoffs between benefits and harms, the Centers for Medicare and Medicaid Services have set an expectation that low-dose CT lung cancer screening orders for heavy smokers must be furnished during a screening counseling and shared decision making visit.[30] Perhaps a similar strategy could be implemented for men facing treatment decisions for a low-risk PCa.

Avoiding treatment complications, particularly related to urinary and sexual function, was a common theme among our subjects. While some men recognized that their cancer may never progress and require treatment, others were more fatalistic. They assumed that treatment was inevitable, but still valued preserving quality of life as long as possible. Numerous studies have shown that quality-of-life concerns are strongly associated with selecting AS.[23, 28, 31, 32] In contrast, men with low-risk cancers who are focused on wanting cancer removed will often immediately select aggressive treatment, thus limiting the opportunity to process information about prognosis and alternate strategies.[23, 24] Volk described how men on AS valued “buying time” to learn about their disease and treatment options while preserving quality of life.[23]

Some men reported being comfortable selecting AS because they viewed it as a systematic monitoring process that could identify progression in time to offer curative therapy. Studies have noted men being uncomfortable with AS because they view it as doing nothing.[22, 24] Our findings suggest that better informing men about the rationale and implementation of AS may help them appreciate that it is an active approach. Our subjects also reported being comfortable with uncertainty, though recognizing that there was a risk that cancer could progress to an advanced stage while on AS and that such an outcome might lead them to regret their decision. Most subjects noted that they trusted their physicians, particularly those recognized as experts in the field, and were very reassured when these clinicians recommended AS.

Our subjects valued the emotional and instrumental support that they received from family and friends but acknowledged sensing some concern about not aggressively treating their cancer. Other investigators have reported that men considering AS, particularly younger men, often receive messages from family and friends that it is a dangerous course.[17, 28, 31] Our subjects felt that family and friends were more supportive when they understood the rationale for AS. These findings suggest the importance of engaging social support from family and friends in the initial treatment decision-making process, including by sharing information about prognosis and treatment options.

In considering adherence to AS, there are two components—deferring AT and continuing with surveillance protocols. Cohort and qualitative studies show that most men switch to AT because there is evidence of disease progression, with fewer men switching due to anxiety.[15] The five subjects in our study who underwent AT based their decision on signs of cancer progression, either PSA or biopsy results, along with a provider recommendation. Others noted that they might consider switching to AT if new and safer treatments became available. Our sample is limited in size, however, none of the men who switched from AS to AT did so because of anxiety or psychological distress/anxiety. A meta-analysis suggested that about 2% of men leave AS annually due to anxiety or other non-clinical reasons.[15] Among the subjects who remained on AS, none expressed much anxiety about their treatment decision. Although some men reported anxiety around the time of surveillance testing, most felt that living with an untreated cancer was not onerous and they did not focus on their cancer between follow-up visits. This is consistent with literature reports, including several systematic reviews, showing that anxiety is generally low among men on AS and decreases over time.[33–36] The randomized Prostate Testing for Cancer and Treatment (ProtecT) trial[37] and observational large observational studies with long-term follow-up, including the Prostate cancer Research International Active Surveillance protocol (PRIAS),[38] the Cancer of the Prostate Strategic Urologic Research Endeavor (CaPSURE)[39], and a population-based study in the United Kingdom[40] found that anxiety is similar between men choosing AS or AT. However, Ruane-McAteer’s systematic review concluded that quantitative observational studies often have important methodological flaws that limit the ability to validly assess the psychological impact of AS. Observational data may suffer from selection bias given that more anxious men may be switching from AS to AT relatively soon after diagnosis.[41] One study prospectively measuring anxiety for 9 months has found higher levels of distress among men on AS than those on AT, which was attributed to men being followed at a center without a strong focus on AS.[42] The absence of anxiety expressed by our contemporary cohort suggests that the benefits of AS are being appreciated while the potential risks are being minimized. Other studies have shown that men made lifestyle changes, including undertaking complementary and alternative medicine treatments, to help them cope with the stress of having an untreated cancer.[21, 24] Interestingly, there was little discussion of lifestyle changes in our sample, just generic mentions of exercise and diet.

There is less literature about adherence with monitoring protocols. Observational data suggest that a substantial proportion of men with a low-risk PCa do not continue with surveillance biopsies following the initial one.[43] This raises concerns that men may miss the opportunity to detect early progression when it is still treatable. Most of our subjects planned to continue with surveillance monitoring. Having regular testing gave them confidence that any changes in their cancer would be detected early. However, men consistently described biopsies as bothersome, causing discomfort and bleeding. Even though none of the patients reported experiencing serious biopsy complications, a few mentioned hoping that MRI testing could replace biopsies due to the invasiveness and physical discomfort of the procedure. Some indicated that they would stop monitoring if they become too old or sick to consider AT.

A theme from our work is the importance of improving the decision-making process. Men and those in their social network could be informed about prognosis and treatment options soon after diagnosis. Although we interviewed men who often sought second opinions when offered only ATs, a more systematic approach to decision-making could ensure that all men understand the range of available treatment options. A shared decision-making process might be the optimal approach, where men make decisions in concert with a clinician based on the best available evidence on the natural history of PCa, their treatment options, and the associated potential benefits and harms of each option. Notably, none of our subjects reported receiving a decision aid, which has been shown to increase knowledge, reduce decisional conflict, and increase engagement in clinician discussions.[44] Importantly, any shared decision-making processes should be implemented efficiently in order to not disrupt busy clinical practices.[45] Enhancing shared decision making for men facing treatment decisions for a low-risk PCa may lead to better clinical outcomes, improved quality of life, and more efficient use of resources.

### Study limitations

There are several limitations to our study. We have a highly selected sample; most men were white, non-Hispanic, and all were being followed at an academic medical center. Therefore, these results may not be generalizable to more diverse populations and/or men treated in community settings. However, several subjects spoke of being rushed into treatment by community physicians, implying that any strategies to better support the treatment decision-making process should be implementable in community practice settings. All of our subjects had followed AS protocols, so we could not determine factors associated with decisions for immediate AT. Our sample included only men who chose treatment after disease progression, so we are not able to report on men who opt out in the absence of progression. Possibly, men experiencing distress might have been less likely to volunteer for our study. Our study had several important strengths. We included men who had completed initial monitoring testing and then either remained on AS or switched to AT. Subjects had remained on AS for an average of 3.8 years and all but two underwent a surveillance biopsy.

### Conclusions

Our cohort of patients being followed at an academic medical center were comfortable selecting and remaining on AS monitoring protocols because they understood that they had a low-risk cancer, wanted to avoid treatment complications, had support from family and friends, trusted their clinician’s recommendation for AS, and were confident that surveillance monitoring would detect early cancer progression. Men who switched to active treatment did so only because their cancer progressed. Men newly diagnosed with a low-risk prostate cancer should be provided sufficient information about prognosis and treatment options, including AS protocols, to make informed decisions. Our findings might not be applicable to men on AS who were not followed at academic medical centers and were surveyed closer to the time of diagnosis.

## Supporting information

S1 Patient Interview Summary (DOCX)
