# Supplementary material for: Why men with a low-risk prostate cancer select and stay on active surveillance: A qualitative study
Source: PLoS One. 2019 Nov 20;14(11):e0225134. doi: 10.1371/journal.pone.0225134 (PMC6867634; doi:10.1371/journal.pone.0225134)
Supplement: S1 Patient Interview Summary — (DOCX) [file pone.0225134.s001.docx]

**AS Patient Interviews Summary**

*Draft 10/12/18*

Patient Experience

- Diagnosis
  - Screening PSA (n=17)
    - Routine PSA screening led to initial concerns/referral for follow up
    - PCP (n=7)
    - Urology (n=9)^[[1]](#footnote-1)^
    - Other (n=1; Cardiologist, PSA as part of cholesterol blood workup)
  - Diagnostic PSA – Urology, after initial screen
    - Few talked about specifically (in contrast to, e.g., 10: “*I went to Dr. XXX …at his first check-up, I had a conversation. Then he did his own PSA test. That came back a little bit higher, even, than the one I had at my general doctor. He suggested a biopsy. We did that.*”)
  - MRI (n= 4) **Initial biopsy?**
  - Biopsy (n=17) **Diagnostic biopsy?**
    - Majority (n=11) discussed biopsy following elevated PSA caught at screening
    - Other/uncertain paths to biopsy
      - E.g., has biopsy after protracted UTI; doesn’t note any other screening/testing
      - E.g., biopsy b/c of family history and BPH
    - No biopsy
      - discusses not remembering biopsy
  - Uncertain (n=1)
    - “*I’m not exactly sure. I, I’ve been going to Dr. XXX for about 10 years now. And, uh, somewhere along he told me that I had cancer, and I almost passed out.*”
- AS conversation
  - Some (n=4) participants were not initially offered AS as an option. These participants had to seek out second opinions, where AS was eventually offered.
    - “*Everybody down there went to the local y, the y that I ultimately went to. They all had, in effect, radiation treatment…. I just thought, that struck me as unusual. In any event, so I did go to the doctor. The number was five-whatever. There was no biopsy, or anything connected with it. I was, all of a sudden, on my road to 40 of radiation, or something. It seemed unusual to me. As far as how could everybody be diagnosed the same and everybody have the same treatment all of a sudden. Never having heard of this, I took time out and I called my next-door neighbor here in [STATE], a retired surgeon. I told him what was happening. He suggested and urged that I hold up whatever I was doing down there, and that I make an appointment with XXX over at [University], and go over and start some study there, the whole issue, okay, which I did.*”
    - “*I think maybe [active surveillance] was mentioned in passing, but it was more like unlike some of the other situations that we’ve gotten into with my wife in that it’s come in and wham, bam, thank you ma’am, and move you out. It’s a foregone conclusion. You got 3 out of the 12. Let’s get at it. Anyway, I just kind of felt pushed and didn’t feel really like I enjoyed working with the urologist.*”
  - Some participants (n=7) reported an AS conversation in which other (treatment) options were not discussed.
    - “*The way it was presented to me in--was that we caught it at such an early stage. That I wasn’t really--it wasn’t really addressed to do it any other way for a time other than just to go on active surveillance, then we’ll see how it goes. …I mean nobody was pushing, or suggesting or anything other than that. …I was completely okay with it because it sounded good to me that the doctor didn’t feel at the time, that I needed to have any treatment on the prostate other than the active surveillance. No, I was--it wasn’t really--well, I knew there were two or three or four, whatever, however many treatments there were cuz I read up a little bit on my own. As far as my interaction with Dr. xxx, and maybe one of his other doctors, there wasn’t any emphasis put on doing anything else.*”
    - “*Well, about as far as treatment goes, there really wasn't any, any treatment about it. Dr. X did say because of--I was younger, I was--see, I was--let's see. Four years--wow, it's four--I was only in my early 50s. Because of my age, he didn't want to--he didn't want--he didn't think surgery was necessary. He said it would be--he said, “I think we'll be okay if we--if we just keep an eye on it and do the surveillance and do”--I think--I believe I went up three times a year that first year doing just PSAs and exams. If we just keep an eye on it. Then we'll give it a year. Then we'd do a second biopsy to see, see if anything had changed, or if anything had spread, or anything like that. That seemed like a good idea to me instead of rushing right into it.*”
  - Others (n=6) had a fuller conversation, addressing both AS and treatment options.
    - “*You know, my wife and I met with Dr. XXX, and he had suggested waiting and watching and seeing ’cause I was still q te young, I was what, 61, 62 years old, and he said, you know, every six months you’ll come in for testing, and we’ll watch it that way. Um, I was really afraid of surgery ’cause I really don’t like the incontinence and other things that can happen. We talked to Dr. XXX about it and he immediately sent me down to—I don’t remember the other doctor’s name. He’s in charge of the CyberKnife surgery. …He met with me for about an hour that day, …Explained to me at that young age he would go for surgery, not CyberKnife ’cause they don’t have a 20 year study yet to what happens to you down the line but, you know, it’s still my choice. I knew it was between that and CyberKnife, and I had done some research and decided to wait.*”
    - “*I discussed with, I guess, a couple of different doctors, the significance of this and the various treatment options and the side effects that I could expect if I did select to have treatment immediately. And it seemed like, although everyone was very clear that, you know, this was my choice, and that even if I elected treatment, that would not necessarily be a bad decision, that the—that there were substantial risks to, to treatment, in the sense that uh, for example, this one doctor put it, “I have patients who fill three type—diapers a day.” Because the surgery uh, caused a certain amount of damage. Okay. So, I knew that there were risks. And I felt pretty comfortable that I was making the rational decision, based on what was known to that time, the probability of the disease progression, the possibility of side effects. And so, I was—I felt pretty comfortable about making the decision to go with the uh, active surveillance.*”
  - For some (n=8), it was unclear how extensive the AS conversation was.
    - “*It may have casually come up to the tune of there a lot of things out there, but right now, what I recommend for you is this active surveillance thing. Of course, based upon my internet research, that had comported with what I had concluded myself.*”
    - “*I didn’t fall off my chair about having cancer or whatever. He said we could look at it in six months and keep an eye on it. He might have said, at one point or another, that there are a lot of ways to treat it other than surgery. I felt that letting six months go, I wasn’t all of the sudden gonna--it wasn’t gonna ravage me and it would be too late. I didn’t worry about that.*”
    - “*Then once they got done with the biopsies and didn’t find anything, well, let’s think about just kind of watching things. …They just weren’t pushing. They were saying, “We can do something if you want. If you’re scared of the word cancer and--” I think that’s what it seems like so many people I’ve talked with, they jump on the bandwagon because it’s cancer.*”
  - No participant remembered decision aid materials being used during the AS conversation, although a few noted being given materials to review (re: treatment options).
  - For 2 participants, the distinction between AS and WW was unclear. Yet both described active surveillance, in terms of scheduled monitoring through PSA/DRE and occasional biopsies.
- Surveillance protocols
  - Timing

| PSA/DRE | MRI | Biopsy |
| --- | --- | --- |
| 6 mo | Annually | Upon recommendation after MRI |
| 3 mo | 2 at diagnosis, unclear after | None |
| 6 mo | 1 | 1 |
| 3 mo | 1 (at NIH) | Annually (“*never off more than a week either way*”) |
| 6 mo | 2 | 1 |
| 6 mo | 2 | 1 (2-2.5 years ago) |
| 6 mo | 2 (at least) | Every 1-1.5 years |
| 6 mo | 2 | Uncertain |
| 6 mo | 1 | 1 fusion biopsy 6 mo after diagnosis |
| 6 mo (1 year once) | N/A | 2, timing uncertain |
| 6 mo – 1 year | N/A | None after diagnosis |
| 6 mo | N/A | 1-2 years |
| 6 mo | N/A | Timing uncertain |
| Timing uncertain | N/A | “I think they’re thinking one or two years for the biopsies” |
| 1 year (just switched from 6 mo) | N/A | 1 since diagnosis, avoids |
| 6 mo | N/A | Annually (upcoming fusion) |
| 6 mo (prior, 3 mo) | 1 | Annually |
| 6 mo | N/A | 1-2 years |
| 4 mo | N/A | Annually (had one, then treated) |
| 3 mo | N/A | 1.5 years (had one, then treated) |
| 6 mo | N/A | 1.5-2 years (fusion) |

- - Testing
    - Participants most frequently monitor with PSA/DRE
    - Participants had an initial biopsy. Several had had one or more follow-up biopsies, occasionally w/MRI, but the schedule for these was much more flexible.
      - “*They’re not on the—no, I’m not scheduled like that*”
      - “*I wouldn’t have entertained [a biopsy] particularly, unless there was an awfully good reason.*”
      - long story about delaying because of personal circumstances
      - “*I would rather not have a biopsy if I don’t need to have one. It seems to me like I don’t need to have one yet again.*”
  - Recommendations/uptake of lifestyle/habit changes, chemoprevention
    - Few participants (n=4 ) noted changes to their lifestyle—in terms of diet, exercise, etc.—as a result of prostate cancer diagnosis and surveillance, at least for a period of time.
      - “*I changed my diet a great deal, did this, did that. …I just took better care of myself.*”
      - “*[W]eather permitting, I walk. I mean I used to walk sporadically. It seems like since I’ve been on active surveillance, maybe psychologically, I’m trying to stay in a little bit better shape maybe.*”
      - “*I did try to cut that [caffeine, alcohol] down, and watch it, and stuff like that. As time goes on, I think because you kind of accept the fact that you've got it, and you don't really--you worry about it a little bit less even though you know in the back of your head you've got it. I didn't probably fall back and, and do what I did before. Didn't really change my life that much. I think the, the initial shock, you change for a little bit. I think you kinda fall back and do what you did before. I think that's probably what I did.*”
    - No changes (n=17 [dietary changes b/c BPH])
  - Indications for stopping AS
    - All participants who had not been treated at the time of interview (n= 16) expected to continue on AS until their provider suggested otherwise. They expected their provider would suggest treatment if their cancer progression warranted it.
    - Aging out (n=2)
      - “*I’d then have to make some decisions because if I was in my 80s I’d say no, I’m not gonna do anything, but at age 65 I still, you know, hope to live at least another 20 years, so I’d have to make some decisions.*”
      - “*Unless I get so old that I don’t think it’s important anymore. That would probably be at least 20 years from now. I intend to live a long time.*”
- Treatment
  - With cancer progression (n=5)
  - Most (n=4) treated with prostatectomy

AS Selection Factors

- Provider role
  - AS conversation
    - Needs to be presented as option
    - Sometimes was the only option presented
  - Trust in physician
    - “*I respected Dr. XXX. He was very highly--he's a department head. He's the department head. And I know my cardiologist, Dr. XXX, had high opinion of him too. He was in the [University] family, so.*”
    - “*Well, I can tell ya up front I have complete trust in Dr. XXX, and I’ve just followed whatever he said, and it’s always made sense to me, despite reading some conflicting information periodically in the newspapers about how this whole area is supposedly overmedicated or over whatever. …I’ve just kind of followed whatever advice he had without any complaints….*”
    - “*I was really afraid of surgery ’cause I really don’t like the incontinence and other things that can happen. We talked to Dr. XXX about it and he immediately sent me down to—I don’t remember the other doctor’s name. He’s in charge of the CyberKnife surgery. …He met with me for about an hour that day, so I thought the people at XXX did a remarkable job of making time and all of that. Explained to me at that young age he would go for surgery, not CyberKnife ’cause they don’t have a 20 year study yet to what happens to you down the line but, you know, it’s still my choice. I knew it was between that and CyberKnife, and I had done some research and decided to wait.*”
    - Faith in doctor, God.
    - Lack of trust when a provider suggests treatment can lead patients to seek other options, such as AS (affected by knowledge) ( n=4)
      - Unimpressed by PCP who did not know about free PSA, disliked a urologist who wanted to do a biopsy, and was unimpressed by another urologist who was about to retire and seemed not to have opened a book in several years.
      - “*Well, when I first went--because my doctor referred me to a local urologist, I went there and had the biopsies. Then due to complications, they gave me to the second in command who usually said you don’t have to worry about anything once she looked at the records and said, “No, you got it. The re lar urologist should’ve seen you.” At that point, she says, “Well, you got 3 out of the 12.” Then I finally got an appointment with him then. It was like line me up for the surgeon. Line me up for the follow-ups for seven weeks, or whatever it is for treatment, and so forth. Just his attitude, I was real concerned about that.*”
- Information sources
  - Provider
    - Sometimes second opinions were solicited ( n=7). In cases where AS was not initially offered (n=4), this second opinion led to the option of AS.
  - Experiences of others (family, friends)
    - Really affected by father’s experience / also, “*I’ve had some folk, some guys in my church, who have had the …brachytherapy, and they came out of it pretty good, but then I had one who has had prostate surgery and it left him with much -- well incontinent and that kind of thing. It’s not something that …I wanted to risk at this time.*”
    - “*I think that’s what it seems like so many people I’ve talked with, they jump on the bandwagon because it’s cancer. I gotta get it out right now. After having had a neighbor who had it done as far as surgery and so forth, he said I wished I had done the watch and wait situation, because I’ve got these complications now and so forth. He said usually you don’t die of it anyway, so as a result, I was more inclined to say let’s watch and wait.*” / Sister also died in surgery, affected his view of surgery as potentially dangerous
  - Research
- Decision making
  - Role
    - Wife and doctors (he thinks) were supportive of AS, sometimes wonders whether it would be better for doctor to say “just do this”—and could blame doctor if it went wrong.
    - Treatment discussion emphasized that it was his decision. Never felt pressured. The right decision is the one that the patient chooses. Notes that people in his church went q ckly to surgery probably without clearly understanding treatment options or complication risks.
  - Decision factors
    - Factor in anxiety when weighting treatment decision
    - Questions whether the positive benefit of reducing anxiety on quality of life are sufficiently emphasized. Have to factor anxiety over a long period of waiting. Having an algorithm where you could plug in numbers to support decision making.
- Surveillance Process
  - Concerns
    - “*I don't think I envisaged any downsides. …Test needs to be done. I mean, I need to shave in the morning or I need to go to the bathroom. That's part of life. At this stage of my life because of certain situations, I need to have certain tests done. …I just go and do it.*”
    - Biopsies uncomfortable (n=5)
    - Needed to pay good attention to it. Some anxiety one to two weeks before testing, didn’t think about it much in between. Good at ignoring things.
    - Has some anxiety, particularly when awaiting PSA and biopsy results. No need to worry about until later.
- Health perspective / Feelings
  - Confident in prognosis and surveillance (MRI, genomic testing)
    - “*I think he’s cautious enough that if he thought we should biopsy it or consider doing something right away, he would’ve said so. I trusted his--and in general, I, I believe prostate cancer, in general, is fairly slow-growing, so it just made sense to me.*”
    - “*When I read the report of the initial finding of the biopsy it gave some percentages and other things and it said that my form of prostate cancer was in the lowest percentage. It was a very tiny, tiny spot, um, and that the degree of growth and metastasizing is extremely, extremely low so I would have had more anxiety opting for surgery or CyberKnife than I did for just waiting. In fact, it was a relief that I could wait when I saw the results.” / “Because of my age and because the chance of this one growing that -- getting so bad in six months was very, very slim so being watched every six months would catch it.*”
    - Confident in urologist (an expert in the field), reassured that cancer “wouldn’t get away from us,” changes wouldn’t happen rapidly with re lar surveillance. Will “deal with it then” if cancer advances in the future. Comfortable on AS, will continue unless cancer changes or he gets too old. Wants to continue re lar check to avoid progression.
  - Tolerant of uncertainty
    - “*Well, reading those PSA scores, that they were going up little by little, Dr. XXX wasn't ready to refer me to Dr. XXX. He made sure I understood that it was going up. And that took a couple of years. So, yeah, I was concerned every time I had that blood exam for cardiology purposes. I was concerned that maybe it gets higher and higher, which was, well, may lead into serious operation. Yeah, I was concerned.” / “Well, I was in the back of my mind that, you know, it's, it's not being treated, it's under control. And obviously the latest PSA score was very encouraging. So that has relieved whatever tension I had. so that's basically where I am, just have to go back to see Dr. XXX back in January. And right now I feel comfortable that we're, we're purs ng at least a solid program here.*”
    - “*I’m not a big worrier. As I said, I felt he was worrying enough, or was careful enough, so that, you know, it was a mild n sance to go see him every six months. Drawing the blood for PSA is not a problem for me with my veins and having the brief hand check, or whatever you call it, by him, and getting the PSA report was not--there wasn’t a lot of anxiety.*”
    - Aware that Gleason and PSA were not 100% accurate, aware of probability of disease progression
- Patient knowledge and beliefs
  - Of cancer as low-risk
    - “*The phrase low-risk was not used, [I: Mm-hmm.] but I realized that Dr. Lynch was very careful to emphasize that this is a very small [I: Mm-hmm. Okay.] area, that this is not to be confused with what patients might know of family history, [I: Mm-hmm.] where somebody has a little spot here, there, or there and then in eight months [I: Yeah.] they're gone.*” P 1/4/15. Aware that PCa can be indolent, but doesn’t want to an ostrich with its head in the ground and ignoring things. Metastasis could begin in 14 to 15 years. Knows he has a very small (micro cells) cancer, but doesn’t want to ignore. Walking away is foolishness. Urologist emphasized cancer is small (did not say low-risk).
    - “*They did the biopsy, and the biopsy came back with a very, very, very small amount of prostate cancer but they did DNA and other type of testing on it, and it was not the type that would normally metastasize….*”
    - Told he had a choice for treatment, comfortable with AS because he was not being told that he had a serious fast-growing cancer and would die very soon without treatment.
    - He’s someone who’s guard is up and doesn’t want to unnecessary treatment (cure is worse than disease). Required confirmation that it was low risk. Has received good explanations about “both sides of it” and is comfortable with AS. Reassured by surveillance results.
    - Confidence in MRI biopsy (enthusiastic about fusion biopsy), but disconcerted when ided biopsy was negative (did my test get mixed up with somebody else?). Hopes MRI could eliminate unnecessary biopsies.
  - Of treatment (side effects)
    - “*I was relieved [I: Mm-hmm] that I didn't have to start treatment, which could well mean surgical intervention or more. [I: Right.] So I was very pleased to hear that.*”
    - “*I was really afraid of surgery ’cause I really don’t like the incontinence and other things that can happen.*”
    - “*I agreed that, while it is alarming to hear the word cancer, I wasn’t really excited about possibilities of doing surgeries that I had heard were not all that successful.*”
    - Felt reassured by AS. Moving in the right direction psychologically. Had read about treatment options. Did not want incontinence. Comfortable with explanation of AS. AS reduces anxiety. Would not like to have surgery. Monitoring can help deal with it earlier. Has faith in the testing.
  - Social responsibility
    - Understood that for Gleason 6 surgery not really recommended; aware of unnecessary surgeries and biopsies. Sees AS as being a responsible decision to reduce healthcare costs.
- Social support
  - Support, esp. from partner, can be useful ( n=14)
  - Don’t involve partners
    - when asked about discussing with wife at time of AS decision, “*Oh, no, no…I mean I didn’t think it was important enough….*”
    - “*I don’t think my wife is really concerned. We really don’t talk about it.*”
  - “*Well, I sought counsel from my wife and my wife only. I didn’t share and I didn’t hide anything from anyone else. It was our business, and we were both there from day one with Dr. XXX.*”
  - “*Well, I had a lot of questions from some of my siblings. …And some of ‘em I know check it out. [I: To be sure you knew what you were talking about.] Exactly.*”
  - “*My wife, I keep everybody involved, because I think if you don’t, you get into a situation where you’re moping. If it’s bugging you, you can talk to somebody about it. Then most likely you can process it and get it out of your system a little q cker, because any time you’re telling your family members cancer, cancer, of course their biggest worry is, “God, is my dad gonna die?” or, “How bad is it, dad?” or to your wife, or brothers, and sisters. Keeping them up to date for me is--it’s not like we talk about it all the time, but it’s like, “Hey, I feel good. There’s nothing going on. Don’t worry.”*”
  - “*Well, my mother and father. They're still with me. They're 86 and 84 right now. They thought it was a good idea. A good move. Because I don't think they ever like to see their children go on the operation table. [I: Right.] My brother. My surviving sister. I had three sisters, but I've lost two of my sisters. She was very supportive. My two daughters were very supportive. At the time, the gal I was dating was very, very supportive. She was standing behind me and, and was worried about me. We were going about it--we were taking care of it, and keeping an eye on it, and they were very, very supportive of, of that. [I: Mm-hmm.] Don't think any of your family or friends wanna see anybody go on--under the knife. It's a--it's a traumatic--it's a big thing. [I: Right.] No doubt about it. I got a lot of support from family and, and my friends, too. My best friends. My, my buddy. I was actually--when I got the call from the doctor, I was over at my buddy M's house. I think when I--when I told him, he was the first person I told. He was--he was shocked. He was bummed. I mean, he thought he was gonna lose his friend. He thought, “He's got cancer. He's gonna die.” [I: Ok.] I think he was probably more shook up about it than I was. A lot of support from a lot of my friends. I was very fortunate to have that.*”
  - Unclear how negative support impacts
    - “*I remember my sister--my, my sister N for the whole time. She just couldn't believe that I was carrying around a cancer. “Get it out. Get it out. Get it out. What're you doing? Get it out. Get it out.” I go, “We're keeping an eye on it.” She was the one person, you know, that was worried about it. My folks weren't so worried about it. They liked the idea of us keeping an eye on it. I remember my, my sister saying, “Get it out. What're you doing carrying around. Just get it cut out.” Then after the second biopsy, and it definitely had--there was another spot, it had spread, I was listening to my sister, N, she was right. My doctor was right. They said, “It's time. Let's not let it go any further. Let's get this thing taken care of. Get it out.”*”
    - “*I think the only person that wonders whether I’ve got a sound of mind is my brother who has had the surgery and everything. He thinks I’m nuts, should’ve had it done ages ago.*”
- Other health considerations
  - Patients can start surveillance because they prioritize other needed treatments (e.g., hepatitis C, other cancers)
    - previously had thyroid cancer (in his 30s)
    - colorectal cancer
    - prior skin cancer
    - “*Now, what actually made me completely change my mind was there was more important matters with my health that were coming about. I had--I have hepatitis C. [I: Okay.] The system had made available for me to get on the proper drugs to remove that. I thought the hepatitis C was more dangerous than the prostate cancer, so that’s what I chose to treat first. That’s what kind of altered it, which I was happy. Now that I’ve actually made that choice, I’m living with that choice a little bit better than I was back then. I was like, oh God, now I’ve got two disorders, and I gotta make a choice of which one’s more important. Well, saving the liver is far more important than the prostate in a light form. Now, if it’s fourth stage, of course you’re in a situation that may have overridden my decision. I think what really did it for me was when I--the doctor and I, we have a pretty good relationship in a sense of patient, doctor. He felt that it wasn’t a necessity to jump on it and have this thing ripped out right away. I kind of took that into play when this other disorder came up.*”
  - Concurrent lifestyle/habit changes, chemoprevention
  - BPH (n=7)
  - Family history
  - Age
    - “*my wife and I met with Dr. XXX, and he had suggested waiting and watching and seeing ’cause I was still q te young, I was what, 61, 62 years old, and he said, you know, every six months you’ll come in for testing, and we’ll watch it that way.*”
- Patient needs
  - Some participants discussed wanting better information (testing) about how aggressive their cancer is. Disappointed that biopsy could not tell whether tumor was aggressive. Would want more information about tumor aggressiveness.
  - Participants thought support groups or other people to talk with might be helpful if they were considering treatment, but not for AS
  - Would welcome detailed interaction with someone going through similar situation. Wants more information to read. Wants to learn more about alternatives. Not sought out support group because it doesn’t seem that urgent. Genetic information—non-painful testing would be fine.

AS Adherence Factors

- Lack of cancer progression (n= 17)
  - “*If the results come back from active surveillance show that more tests need to be done, and those tests indicate that the cancer has started to grow then I would—then it would be a different situation. I’d then have to make some decisions because if I was in my 80s I’d say no, I’m not gonna do anything, [I: Mm-hmm.] but at age 65 I still, you know, hope to live at least another 20 years, so I’d have to make some decisions*”
  - Expects to continue AS, but would consider AT if he developed pain or dysuria. Would consider radiation, but only if recommended. Wonders whether aggressive treatment would insure him some kinda quality as he ages or if it just experimental stuff.
  - On what would make him consider switching, “*We do this next biopsy and they strike some cores, and I get a Gleason score of seven or eight.*”
- Other Symptom Progression (BPH or other urinary symptoms)
  - “*I’m not having any kinds of issues that can normally happen with prostate cancer. …I’ll just keep watching and waiting.*”
  - “*Anyhow, I haven’t, as far as repercussions, or I only take one dose of Flomax. I have a--my prostate is enlarged. I guess it’s a large prostate, so I have to--I was having a real issue with urination and being in difficult to urinate. …I guess the--that’s about what I can think of right now as far as to comment on. …I did go, I know doctor, I did go to a seminar three or four months ago at [University]. It had to do with enlarged prostate. That’s what it was zeroing in on different treatments. I guess you’d say if I was to have--if my prostate got to a point where I couldn’t urinate with the Flomax, what would we do to alleviate that issue?*”
  - “*if there--to me, if something shows up that changes how things--or I may not able to urinate, feel like it’s causing problems that way, and be concerned, and head back to [CITY] and have them check things out.*”
- Provider role
  - Trust in physician (n=5)
    - “*Not unless Dr. XXX and folks at [University] suggested it, that you’d better change your course of action because things aren’t goin’ in the right direction.” / “No one can exactly tell that [what is significant progression], except that I think that the combination of folks there at [University]…. …You feel like they’re the professionals. They’re the experts. They had the experience. The place has a reputation.*”
    - “*I feel like I’m being surveilled well by (laughs) Dr. xxx group. I feel like they’re on top of it.*”
    - “*Until the doctor tells me that I’m wrong not to continue doing the active, then I’m--I have no sense internally or chronologically that it would be time to do something different.” / “A recommendation from the doctor and not just for what you think recommendation, but I think we better do something soon. …All three doctors, but particularly the last two, I felt comfortable talking with the doctor about this and I felt that the doctor knew as much as he could know about what was going on inside of me as opposed to not knowing or not caring or not being too busy or something, which probably wouldn’t be a good evaluation from my doctor-patient relationship. (Laughs) I felt very comfortable with both of the doctors and they would--they are on top of this maybe more than I am. If they push me into it, I would probably accept, I just would not be anxious to do it right away if I didn’t have to.*”
  - Providing information
    - “*I think the doctor treating you, and whoever would he assign, should be able to explain in detail the facts of what that means and which percentages of reoccurrence, and things like that.*”
- Health perspective / Feelings
  - Confident in prognosis and surveillance
    - “*Well, I'm glad we're under active surveillance, because that means, if there's any change, he would know quickly. I think it sort of means they will know as soon as possible.*”
    - “*I feel very confident in the approach that I’m on with Dr. XXX in [University]. … I suspect that if there’s trouble ahead, that someone’s gonna discover it in a timely fashion, and then we’re gonna initiate corrective action at that point in time.*” P 14. Believes it prudent to monitor closely, you’re going to see if the train is still on the tracks. Recognizes that surveillance does entail certain risks.
    - “*As long as I know there’s still cancer in there and as long as it isn’t bothering me or being a high-risk situation, I have no intent whatsoever to change.*”
    - Urologist described AS protocol, rational of preserving quality of life—particularly because he was younger man. Reassuring that he would be keeping a really good eye on it.
  - Tolerant of uncertainty (lack of anxiety)
    - Asked about anxiety with monitoring, “None whatsoever. …Nothing at all.” / “Mine wasn’t that big a deal. If, if those results had come back and said it is the type that metastasizes, and we still did active surveillance, yeah, I’d be worried and stuff like that” DNA results reassuring.
    - “*No. It’s something that I have not. I don’t know why because it would seem natural you would. I guess I feel like I’ve been lucky, that I’ve been able to maintain this being on the active surveillance. I haven’t had the bad, so to speak, the bad news so far. Every time I go in for a checkup, I have a little anxiety thinking about it and that day, not knowing what’s gonna happen. What diagnosis I’m gonna get. I can’t say that I’ve had much of an anxiety issue with it, frankly. Because I just feel like I’m a little bit lucky.*”
    - While on AS worried about it growing, can’t dwell on it. Aware of men living 20+ years with untreated prostate cancer.
  - Information seeking
    - “*The two of us have a tendency to go to a doctor and ask a heck of a lot of questions. What’s gonna happen? Why, and so forth? Everybody we know seems to be--including our kids seem to be--they walk in like cattle to a slaughter house and get--don’t ask questions, don’t do nothing. We both ask a lot of questions…*”
- Patient knowledge and beliefs
  - Of cancer prognosis
    - “*There were two separate, young’ish doctors there I assumed [I: Mm-hmm.] that were working with Dr. XXX. [I: Oh. Okay. Uh-huh.] This XXX, now I visited with at least twice. [I: Uh-huh.] Prior to that, it was another young’ish doctor, who may have been, in fact, a little bit older than XXX. [I: Mm-hmm.] They were both after the biopsy, in any event, when I first met these ys. [I: Uh-huh, okay.] They both told me, in effect, that well, three or four little things here that were down someplace in the biopsy were--if you had to have this, this was the best possible place to have it, [I: Uh-huh.] and the type, whatever it was. Not to worry. Bottom line.*”
  - Of what information tests provide
    - “*I don’t know anything about this case as far as how rapid things accelerate, but in my own mind, I guess, I’m not particularly alarmed, concerned, disturbed. I mean, I had a biopsy 18 months ago. I had this MRI in December of last year, ten months ago. Those things, which to me, I’m attaching a lot more significance to, maybe unnecessarily so, than those PSA test numbers. Recognizing again, and again maybe I’m factoring too much in, that for neither test did I particularly--You know? I guess that--and again, I’m convinced my, my knowledge gleaned on the internet --that sexual activity, while it drives up PSA, might not or does not suggest that the particular person with a PSA elevated has prostate cancer.*”
    - Very small amount of cancer on biopsy. Had DNA testing that showed it was not the type that would normally metastasize. Aware that it was tiny spot, low risk of spreading. Reassured that waiting was best option, slim chance of getting bad in six months. DNA results reassuring—would be worried about being on AS if DNA tests said cancer likely to metastasize.
  - Of treatment & side effects
    - “*I don't like surgery. There is no minor surgery. There's only surgery that's less invasive than something else.*”
    - “*It’s the side benefits or the side effects that concern me from the surgery perspective. I guess one more comment is, I don’t know if it’s possible to have a perfect surgery. I’m concerned that I won't have the perfect surgery.*”
    - AS is right choice because cancer’s not bad enough to warrant stripping out. Worried about not having a good quality of life which can happen is something bad happens in the surgery.
  - Of treatment inevitability
    - “*I mean, in the back of my mind I knew that I would have to address it in some way. I knew that. Yeah.” / “You know you’re gonna have to treat it at some point, or you should. You know, you’re gonna have to deal with it.*”
    - “*I felt there will be a day that would come sooner than it has come so far, where the doctor would say, “It’s time to do something more than just monitor this thing with a meeting, a discussion, every six months to a year and a rectal examination maybe even more than a biopsy. Maybe whatever else will be next.*”
  - Of treatment advances
    - “*And my idea was to, hopefully, to ride this out to get a real good and easy treatment for prostate cancer. Meaning [I: Mm-hmm.] I started readin’ stuff like the ultrasound stuff. [I: Mm-hmm.] And then, you know, some of the radiations and different things seemed to be really comin’ on board, you know, [I: Mm-hmm.] that I could ride it and get somethin’ that would be real easy that would save me from the three I’s. [I: Yes.] So [I: Uh-huh. Okay.] that was really my idea. [I: Mm-hmm.] So, you know, for three years I was able to, [I: Yes.] three plus years I was able to do that.*”
    - “*The gold standard is to have your prostate jerked out. Well, I'm sorry, I'm not a gold standard person. I know there's other options out there, and the way the medical field is changing. You know, part of the reason, I fi re if I can get by an active surveillance without it killing me, God knows what they're gonna come up with six months from now, where, you know, they're making advances every day. Maybe they'll--I have a good friend who was diagnosed stage 4 malignant melanoma. He's going through immunotherapy up here. Last PET scan, he has nothing. Well, it used to be stage 4, you know, you're just death. They're making advances all the time. Maybe they're gonna come up with a pill I can take and shrink the prostate and cure the cancer, I don't know.*”
- Surveillance
  - Ease of process / Routine nature
    - “*I just got comfortable with the security of being on surveillance. …I just got to the point where that’s not a major factor. There was no depression from that, or concern or worry about it. In the beginning everything I--once I found out I had cancer, every problem I felt was related. I’d get a sore shoulder or whatever—so you’re very concerned. I’m out of that stage now.*”
    - Hopes MRI could eliminate unnecessary biopsies. P 9. Would welcome new testing to find cancer—nano-particles with MRI—to avoid biopsies and tests that could better determine whether cancer is aggressive.
  - Active
    - “*I feel very positive about doing it. I don't think it is exactly, but I almost consider it a treatment. It's almost like a treatment. It's not that it's actually intervening in some way. …[But] based on the biopsy, we'll treat it accordingly. I feel this is kind of like that, that this is part of the treatment. Any further treatment or surgical treatment would just be another step on the same rail. …I mean, what's happening to me might be mostly diagnostic in the true sense of medicine, but from the patient's standpoint, I'm considering it part of the modality. This is what you do in order to remain healthy.*”
  - Flexible schedule
    - Patients and providers determined an adherence schedule (6 mos, 1 year, etc.) that works for them. Often can be flexible, esp. in terms of delaying biopsy.
  - Rigorous schedule
    - “*I think active surveillance should be more than just a PSA check. I think that once a year, the things that--or you need to do an MRI or a CT scan. Of course, a PSA should be done. That should be routine while you’re on surveillance. I’m not sure what Dr. XXX has planned for me next other than, “Come back for your next visit.” That really hasn’t been--I thought even this time they may do another MRI or something like that.*”
  - Lower discomfort with testing, esp. biopsy
    - “*Well, it’s not something I love to try to do, but it was a situation where with this last round that they said, “Well, this way we can confirm where it is and so forth.” It was a little bit more reassuring in that we know where we’re trying to shoot, and we’re gonna try to shoot in this particular place. Then it was neat for being able to get up off the table and have doc show me the screen and say, in living color just about, “Well, this is where it went, and this is how deep it went.”…*”
- Health considerations
  - Lower urinary tract (BPH) symptoms and control
  - Other issues
    - “*In a certain sense, the prostate is so far away, honestly, it's—[I: It's the least of your problems?] Yeah. Yeah. The real problem is why won't that sore on my leg heal? When are we gonna get rid of that red and flaking and all the little venal valves seem to be bulging?*”
  - Age
    - “*When I looked at an evaluated active surveillance course, [I: Mm-hmm.]contrasted against—and where I was, I thought, and the doctor seemed to confirm with me, and again going back to the numbers that’d always been until I got to 70 or 72, [I: Mm-hmm.] were in a safe range. Knowing that they tell you maybe if you’re 75 or 80, that if that PSA number goes up to 8, you’ll still go back to “You’ll die with it, not from it”.*”
    - “*If the results come back from active surveillance show that more tests need to be done, and those tests indicate that the cancer has started to grow then I would—then it would be a different situation. I’d then have to make some decisions because if I was in my 80s I’d say no, I’m not gonna do anything, but at age 65 I still, you know, hope to live at least another 20 years, so I’d have to make some decisions.*”
- Experiences of others
- Social support
  - “*She makes sure I actually go for the tests every six months. …Then, as soon as the letter comes in the mail, I have to rip it open immediately.*”
  - “*She has a spreadsheet. She thought it was important for us to have a spreadsheet and monitor our results of all our examinations. [I: Mm‑hmm.] Our blood pressure, our heart rate, or whatever else we have. She has a spreadsheet and we have the PSA on it. She says, “Okay, what’s your PSA?” I tell her. What’s my blood pressure and all that and so I tell her and she posts into a Spreadsheet. It seemed like before my next appointment, she pulls that out and says, “Okay, here’s--have this in your head.” Maybe I suggest that we pull it out, but anyway together. If I don’t say something to her, she will say something to me before my appointment. This is your trend right now. Keep in mind tomorrow or the next day when you go in how it relates to what we have seen in the past. She’s very assertive in that area.*”
  - “*My family and friends have all been supportive of my decision. We don’t really talk about it anymore, because it’s just something I do every six months or every year and the next day, things are same old same old. They don’t bother me about it. They’ve supported what I decided, and so life goes on.*”

Regret

- Treatment selection
  - Not certain if he would make same decision again, aware that even when you make best possible decision based on the best possible information things can go wrong. Would put more weight on taking action than watching and waiting. Time machine perspective is that there was not anything that the doctors should have done differently.
  - Could see regretting decision if cancer spreads, but describes AS as a “wait-and-see thing” and he doesn’t spend a lot of time thinking about it.
  - After 2nd biopsy showed progression all agreed it was time to get it out. No regrets about AS—good idea, kept quality of life for the next year and a half.
  - Feels lucky to have gotten another two more years before surgery. Important not to go through surgery before you had to. Glad that he could hold it off as long as he could.
  - Not anxious about appointments, not second guessing, suspects that if there is trouble someone’s gonna discover it in a timely fashion.
  - Would have continued AS without qualms if not progressing. No regrets. I don’t feel like I could have really done anything different.

Reasons for Choosing Treatment

- Cancer progression
  - (n=4)
- Patient knowledge and beliefs
  - Symptoms from enlarged prostate (Somewhat uniquely, patient XX has a very clear sense (for a patient) of these symptoms and effects from prostate cancer as distinct)

Recommendations

- Support Group
  - “*I think for those who are very concerned, especially those who are experiencing surgery or the eq valent, I think it [a support group] would be good. I think that would be very good, because they can speak to one another about the problem and share experiences, medication, and I think that could be helpful to them. At this point I don't--I'm not, how should I say, willing to jump into that type of conversation, I guess, with others.*”
- Recognizing importance of screening to patients
  - “*I don’t know where you’re headed with this or what everybody’s tryin’ to prove, but when I read those articles about--and I’ve discussed them with Dr. XXX--that, you know, about how this is a waste of time and monitoring PSAs. Now, the biopsies are, are extremely uncomfortable. I can tolerate, but the monitoring PSA and whatever, I read articles about how this is overdone. It’s a waste of time, and all I can think of is, you mean people like me should just go ahead and die whenever. It, it, it just--I don’t understand the articles, except-- or the thinking behind the articles, except that it’s purely statistics. It’s like, you know, 90 men would have never needed this, but if I’m one of the other 10, which I found out I am now, it--so I never understood that.*”
- More information in surveillance / better tests
  - “*I think it’s important though, when you conduct these--I mean it’s important if you’re going into surveillance that your doctor explains to you after each of the check-ups [I: Sure.] especially the positive results about, “That was good. Things are stable and we’ll see you in another year,” and things like that. Obviously, you’re always worried. You know you have cancer. [I: Sure.] You’re not sure what the percentages of it that spread and don’t spread, and things like that.*”
  - “*Well, like I said, if they’ve got a way of checking and saying, “Hey, your cancer is the aggressive kind. We ought to go in now before it gets worse,” then I’d probably go for that just to say that.*”
- Anxiety

1. “It’s been a while now…I guess we did the blood work.” / No specific discussion in interview of PSA exams, but had been seeing urology for several years for BPH [↑](#footnote-ref-1)
